# Real-time laser speckle contrast imaging for intraoperative neurovascular blood flow assessment: animal experimental study

**DOI:** 10.1038/s41598-023-51022-2

**Published:** 2024-01-19

**Authors:** Anton Konovalov, Fyodor Grebenev, Dmitry Stavtsev, Igor Kozlov, Vadim Gadjiagaev, Gennadii Piavchenko, Dmitry Telyshev, Alexander Yu. Gerasimenko, Igor Meglinski, Savely Zalogin, Anton Artemyev, Grigorii Golodnev, Tatiana Shumeiko, Shalva Eliava

**Affiliations:** 1 Burdenko Neurosurgшcal Center, Moscow, Russian Federation; 2 Institute for Bionic Technologies and Engineering, I.M. Sechenov First Moscow State Medical University, Moscow, 119991 Russian Federation; 3 Institute of Biomedical Systems, National Research University of Electronic Technology, Zelenograd, Moscow, 124498 Russian Federation; 4 Department of Human Anatomy and Histology, I.M. Sechenov First Moscow State Medical University, Moscow, Russian Federation; 5 College of Engineering and Physical Sciences, Aston University, Birmingham, UK; 6 Department of Operative Surgery and Topographic Anatomy, I.M, Sechenov First Moscow State Medical University, Moscow, Russia

**Keywords:** Neuro-vascular interactions, Neurological disorders

## Abstract

The use of various blood flow control methods in neurovascular interventions is crucial for reducing postoperative complications. Neurosurgeons worldwide use different methods, such as contact Dopplerography, intraoperative indocyanine videoangiography (ICG) video angiography, fluorescein angiography, flowmetry, intraoperative angiography, and direct angiography. However, there is no noninvasive method that can assess the presence of blood flow in the vessels of the brain without the introduction of fluorescent substances throughout the intervention. The real-time laser-speckle contrast imaging (LSCI) method was studied for its effectiveness in controlling blood flow in standard cerebrovascular surgery cases in rat common carotid arteries, such as proximal occlusion, trapping, reperfusion, anastomosis, and intraoperative vessel thrombosis. The real-time LSCI method is a promising method for use in neurosurgical practice. This approach allows timely diagnosis of intraoperative disturbance of blood flow in vessels in cases of clip occlusion or thrombosis. Additionally, LSCI allows us to reliably confirm the functioning of the anastomosis and reperfusion after removal of the clips and thrombolysis in real time. An unresolved limitation of the method is noise from movements, but this does not reduce the value of the method. Additional research is required to improve the quality of the data obtained.

## Introduction

The use of various blood flow assessment methods in neurovascular interventions is crucial for reducing postoperative complications^1,2^. In the realm of intraoperative assessment of cerebral arterial blood flow during neurosurgical procedures, conventional ultrasound diagnostic modalities, namely, contact Dopplerography and flowmetry, have long been utilized. Contact Dopplerography, although a cost-effective and easily replicable method, is inherently characterized by manual operation, thereby introducing a measure of inaccuracy. The precision of its outcomes is contingent upon various factors, such as the angle of insonation, the point of examination, and the thickness of the vessel, rendering it a qualitative rather than quantitative technique^2^.

It is imperative to underscore the accessibility and economic viability of contact Doppler sonography, notwithstanding its limitations in appraising the patency of diminutive perforating arteries, the integrity of which significantly influences neurological outcomes. Furthermore, the susceptibility of Doppler sensors to damage and their unsuitability for frequent sterilization have been substantiated through extensive years of clinical experience^3^.

In contrast, ultrasound flowmetry stands out for its capacity to measure the volumetric velocity of blood flow, providing a qualitative departure from the linear velocity delineated by Dopplerography^2^. This attribute renders flowmetry an indispensable tool for assessing blood flow in anastomoses and for detecting the occlusion of large vessels in the context of aneurysm trapping, which is particularly pertinent in patients with large and giant aneurysms.

The advent of indocyanine green angiography (ICG) has captured the attention of vascular neurosurgeons as a technically uncomplicated and noninvasive vascular imaging modality. However, the technique is not devoid of limitations, as the surgeon is restricted to tracing fluorescent blood flow solely within immediate visibility, a constraint exacerbated by occlusions caused by vessel branches, clips, or aneurysms, among other obstructions^4^.

To address these limitations, the application of multispectral imaging (MFL) for fluorescence in the intraoperative field under white light mode has been introduced^5^. This innovative technology enables concurrent visualization of ICG fluorescence and white light, facilitated by optical filters incorporated into the microscope and specialized software amalgamating both images. Consequently, real-time imaging of the fluorescence effect is achieved, affording the surgeon an ongoing view of both anatomical structures and the dynamic fluorescence within vessels.

Notwithstanding the aforementioned modalities, contemporary multimodal operating theaters, such as VISIUS and AMIGO, integrate magnetic resonance angiography (MRA) and computed tomography angiography (CTA). While these techniques offer comprehensive vascular imaging, their requisite preparation time and the duration of the examination within the confines of the operating theater pose logistical challenges, precluding concurrent surgical interventions during the imaging process^6^. However, there is a continuous contactless method that can assess the presence of blood flow in the vessels of the brain without the introduction of fluorescent substances throughout the intervention.

Laser speckle contrast imaging (LSCI) is a promising method for intraoperative blood flow assessment during neurosurgical intervention^7^. The LSCI calculates an indicator called the speckle contrast, which is quantitatively related to the velocity of light scattering particles in a given field^8^. This allows us to determine the relative velocity of blood flow in each pixel of a given field and create a colour image, the intensity of which will characterize the relative velocity of blood flow. Many studies have confirmed the effectiveness of this method in assessing brain perfusion in animals^9–11^. However, the number of neurosurgical surgeries is limited to only a few series of observations^8,12^. One of the limitations of this technique until recently was the postprocessing of the obtained data, which requires an actual amount of time^11,13^. The aim of this work was to evaluate the effectiveness of the real-time LSCI method for assessing blood flow in standard cerebrovascular surgery situations involving the following common carotid arteries: proximal occlusion, trapping, reperfusion, anastomosis, and intraoperative vessel thrombosis.

## Results

### Assessment of blood flow in stenosis, proximal occlusion and CCA trapping

**Figure 1:**
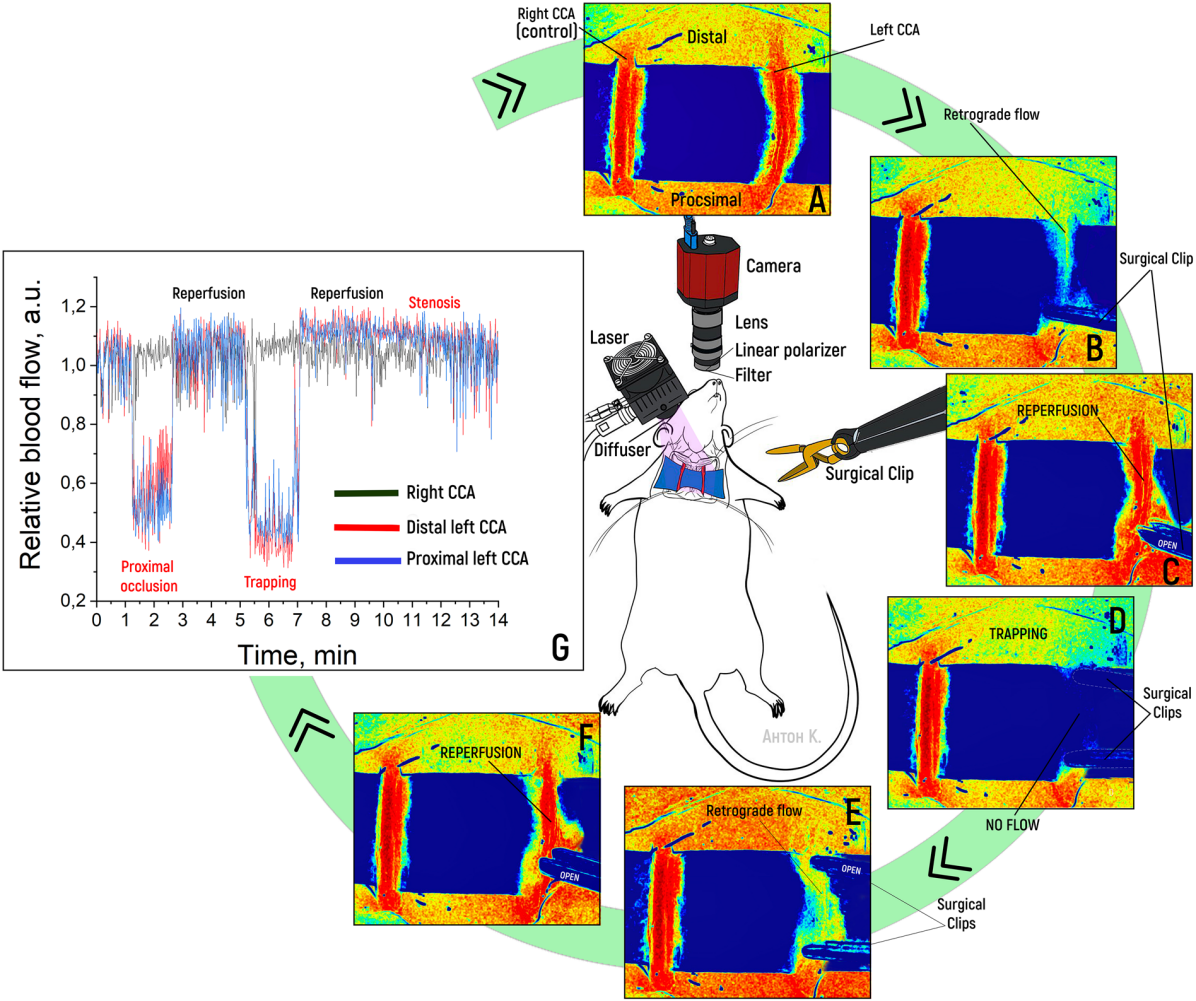
Sequential measurements of the parameters of vascular blood flow in the common carotid artery using the LSCI system, in order: normal blood flow (**A**), blood flow under conditions of proximal occlusion (**B**), restoration of blood flow (**C**), blood flow under conditions of bilateral clamping of the artery by vascular clips (**D**), restoration of distal blood flow (**E**), complete recovery after vessel trapping (**F**), and real-time graph of blood flow dynamics at two points in the right and left CCAs (control) (**G**).

In this study, using the real-time LSCI method, it was possible to verify a moderate decrease in blood flow in the distal part of the left common carotid artery (CCA) against the background of stenosis and a pronounced decrease in proximal occlusion. Additionally, using this method, it was possible to identify the presence and intensity of retrograde blood flow against the background of proximal clipping, which could not be achieved with contact Doppler sonography. Otherwise, there was a complete correspondence between the decrease in blood flow according to LSCI and intraoperative contact Doppler ultrasonography. During trapping, the method made it possible to detect the absence of blood flow in the switched-off segment of the blood flow and thus differentiate minimal retrograde blood flow from the absence of blood flow. When the clips were removed (after proximal occlusion), the method revealed that blood flow was restored to the previous level, with a slight increase in perfusion. The LSCI (a.u.) of the left CCA was assessed distal and proximal before and after clipping and following reperfusion, trapping and reperfusion. The mean value of the LSCI initially left CCA distal was 26.23 a.u. (± 2.24), which decreased to 15.26 (± 3.29) after proximal clipping (−58.1%). There was obvious retrograde flow at the distal part of the left CCA (Fig. 1). During reperfusion, the distal left CCA rose to 26.92 a.u. (± 2.13). After trapping, the flow rate decreased to 12.12 a.u. (± 3.81), and no retrograde flow was observed (Fig. 1D). Reperfusion after trapping showed an increase in the LSCI up to 27.97 a.u. (± 1.72), which was the complete restoration of blood flow. The experimental data are presented in Table 1.

**Table 1:** LSCI values based on the results of the experiment involving proximal occlusion, trapping and stenosis of the CCA.

	Mean value (standard deviation)	Type of distribution (Kolmogorov‒Smirnov value)	Friedman’s Chi-square value	*p*
Initially, right CCA	28.16 (± 2.16)	Non-normal (0.16)	31.63	< 0.001
Proximal clipping (of left CCA) right CCA	28.93 (± 1.27)	Non-normal (0.15)		
Reperfusion 1 right CCA	28.27 (± 2.27)	Non-normal (0.11)		
Trapping (of left CCA) right CCA	29.38 (± 2.53)	Non-normal (0.25)		
Reperfusion 2 right CCA	29.20 (± 1.38)	Non-normal (0.05)		
Initially, left CCA distal	26.23 (± 2.24)	Non-normal (0.18)	228.19	< 0.001
Proximal clipping of left CCA distal	15.26 (± 3.29)	Normal (0.05)		
Reperfusion 1 left CCA distal	26.92 (± 2.13)	Non-normal (0.19)		
Trapping of left CCA distal	12.12 (± 3.81)	Non-normal (0.18)		
Reperfusion 2 left CCA distal	27.97 (± 1.72)	Non-normal (0.17)		
Initially, left CCA proximal	26.20 (± 1.80)	Non-normal (0.13)	224.76	< 0.001
Proximal clipping of left CCA proximal	13.67 (± 2.33)	Non-normal (0.13)		
Reperfusion 1 left CCA proximal	27.27 (± 2.30)	Non-normal (0.19)		
Trapping of left CCA proximal	13.25 (± 3.16)	Non-normal (0.20)		
Reperfusion 2 left CCA proximal	28.00 (± 1.83)	Non-normal (0.19)		

**Figure 2:**
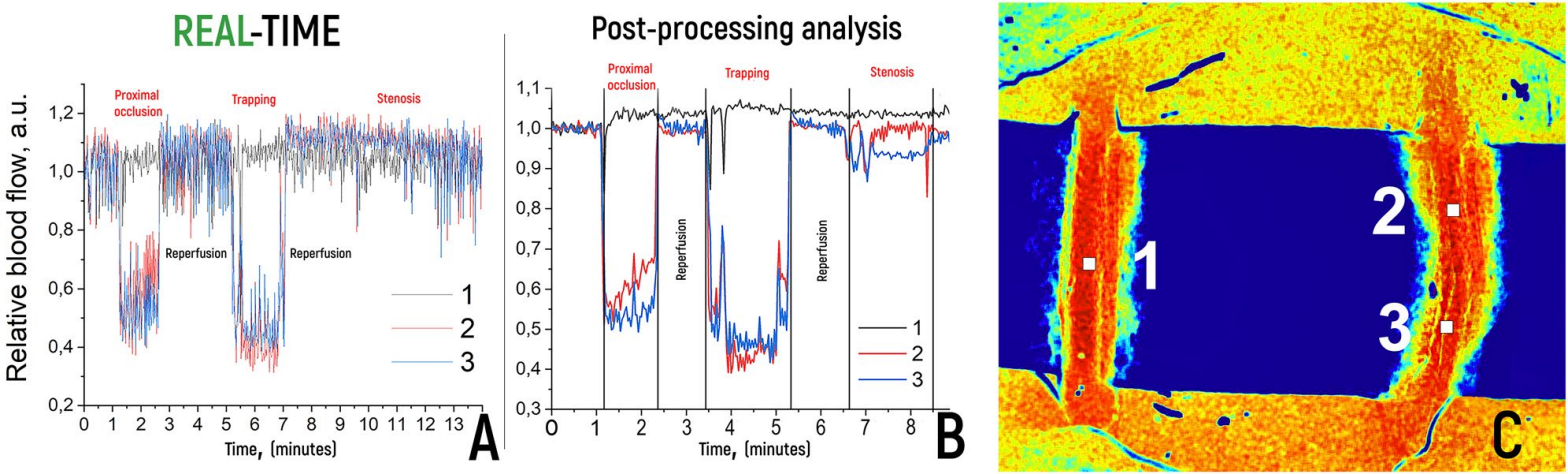
(**A**) Graph of measurements of relative blood flow during real-time LSCI. (**B**) Graph of blood flow dynamics during postprocessing of LSCI data with noise suppression from movements, with the same ROI. C—ROI at two points in the left CCA (2, 3) and the right CCA (1—control).

The experiment was then performed in postprocessing mode to evaluate the differences in the resulting data. As shown in Fig. 2, the postprocessed LSCI plot provides better visualization and sensitivity to changes in blood flow parameters. However, as one can see in real-time, one can reliably and qualitatively observe a decrease or absence of blood flow, as well as reperfusion, which is acceptable for intraoperative evaluation of blood flow.

### Evaluation of blood flow during vascular microanastomosis

**Figure 3:**
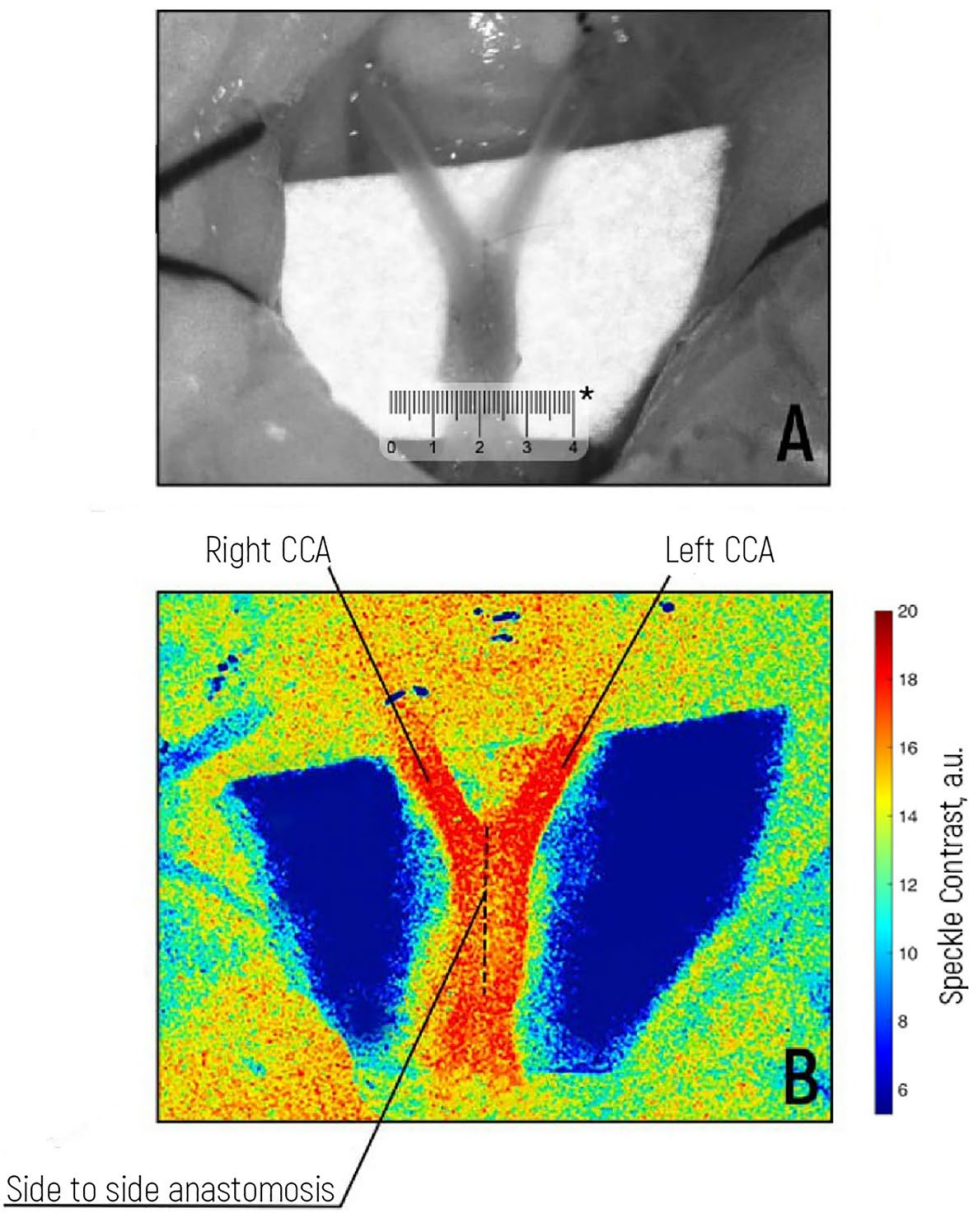
(**A**) Intraoperative view of a functioning side-to-side anastomosis. Creating a side-to-side anastomosis between the right and left common carotid arteries: A midline incision was made in the neck, carefully dissecting through the subcutaneous tissue to expose the underlying muscles. The common carotid arteries were identified and isolated bilaterally. The arteries were then dissected from the surrounding tissues, ensuring minimal damage. Using microsurgical instruments, a longitudinal arteriotomy was performed on both common carotid arteries. The arteriotomy edges were aligned precisely, ensuring optimal apposition and avoiding overlap or gaps. The anastomosis was then performed by placing anchor interrupted sutures and running sutures using a fine microsurgical needle and nonabsorbable 10-0 nylon suture material. Appropriate tension was maintained to achieve secure closure without compromising blood flow. (**B**) LSCI visualization of blood flow in the anastomosis. *The scale bar is 4 mm.

In this study, the RT-LSCI method made it possible to detect the patency of the side-to-side anastomosis between two CCAs (Fig. 3). With proximal exclusion of the left CCA, the distal parts of both CCAs were satisfactorily filled from the right CCA. The RBF in the distal part of the left CCA corresponded to the normal value measured before anastomosis. Notably, the suture (Prolene 10/0) did not interfere with the LSCI (see Fig. 3B).

After creating an anastomosis, a decrease in blood flow according to the LSCI was correlated with the contact Doppler imaging data (Table 2). The mean linear blood flow velocity (LBFV) of the left CCA proximal to the mean (standard deviation) was initially 32.79 cm/sec. (± 3.30), which decreased to 12.08 (± 2.86) after anastomosis. There was a 63% decrease in flow velocity, but the LSCI of the left CCA proximal to the anastomosis increased up to 14%. The LBFV of the left CCA decreased by 34% after anastomosis. The LSCI of the left CCA distal to the anastomosis increased by 32% (from 10.19 to 14.48 a.u. (± 0.56)). The LBFV of the right CCA distally was initially 39.54 (± 3.172), which decreased after anastomosis. The LSCI of the right CCA distal to the anastomosis increased to 28.27%. According to Bernoulli's law of hydrodynamics, the total mechanical energy of a fluid that flows smoothly, which includes pressure, kinetic, and potential energies, remains constant. This means that when the speed of a fluid increases, the fluid’s pressure or potential energy decreases simultaneously. In simpler terms, a fluid that flows slowly exerts more pressure than a fluid that flows rapidly. A decrease in the velocity of anastomosis indicated an increase in diameter and flow volume. Our LSCI data are in alignment with Bernouslli’s law of hydrodynamics. The LBFV of the right CCA proximal to the initial site was 39.54 cm/sec (± 3.17), which increased to 66.78 cm/sec (± 7.84) after anastomosis. However, the LSCI of the right CCA proximal to the anastomosis also increased by 21.3%. This could be caused by suture mechanical stenosis or surgical manipulation of the vessel and may also be a haemodynamic reflex response after reperfusion or deviation.

**Table 2:** Dynamics of Doppler (LBFV) and LSCI before and after side-to-side anastomosis between the CCAs.

	Mean value (standard deviation)	Dynamics of the average value in percents	Statistical criterion and its value	*p* value
LBFV, left CCA, proximally, initially (cm/sec)	32.79 (± 3.30)	−63.15%	Wilcoxon Test (Z) = −5.115	< 0.001
LBFV, left CCA, proximally, after anastomosis (cm/sec)	12.08 (± 2.86)			
LSCI left CCA proximally initially (a.u.)	10.88 (± 0.16)	+ 14.02%	Paired Student t Test (T) = −7.604	< 0.001
LSCI left CCA proximally after anastomosis (a.u.)	12.41 (± 1.14)			
LBFV, left CCA, distally, initially (cm/sec)	32.79 (± 3.30)	−34.44%	Wilcoxon Test (Z) = −4.403	< 0.001
LBFV, left CCA, distally, after anastomosis (cm/sec)	21.5 (± 1.25)			
LSCI left CCA distally initially (a.u.)	10.93 (± 0.19)	+ 32.52%	Wilcoxon Test (Z) = −4.703	< 0.001
LSCI left CCA distally after anastomosis (a.u.)	14.48 (± 0.56)			
LBFV right CCA, proximally, initially (cm/sec)	39.54 (± 3.17)	+ 68.90%	Wilcoxon Test (Z) = −4.640	< 0.001
LBFV, right CCA, proximally, after anastomosis (cm/sec)	66.78 (± 7.84)			
LSCI right CCA proximally initially (a.u.)	10.65 (± 0.16)	+ 21.34%	Wilcoxon Test (Z) = −4.703	< 0.001
LSCI right CCA proximally after anastomosis (a.u.)	12.93 (± 0.79)			
LBFV right CCA, distally, initially (cm/sec)	39.54 (± 3.17)	−54.09%	Wilcoxon Test (Z) = −4.223	< 0.001
LBFV right CCA, distally, after anastomosis (cm/sec)	18.15 (± 2.74)			
LSCI right CCA distally initially (a.u.)	11.13 (± 0.20)	+ 28.27%	Wilcoxon Test (Z) = −4.703	< 0.001
LSCI right CCA distally after anastomosis (a.u.)	14.28 (± 0.59)			

### Evaluation of blood flow in intraoperative thrombosis patients

**Figure 4:**
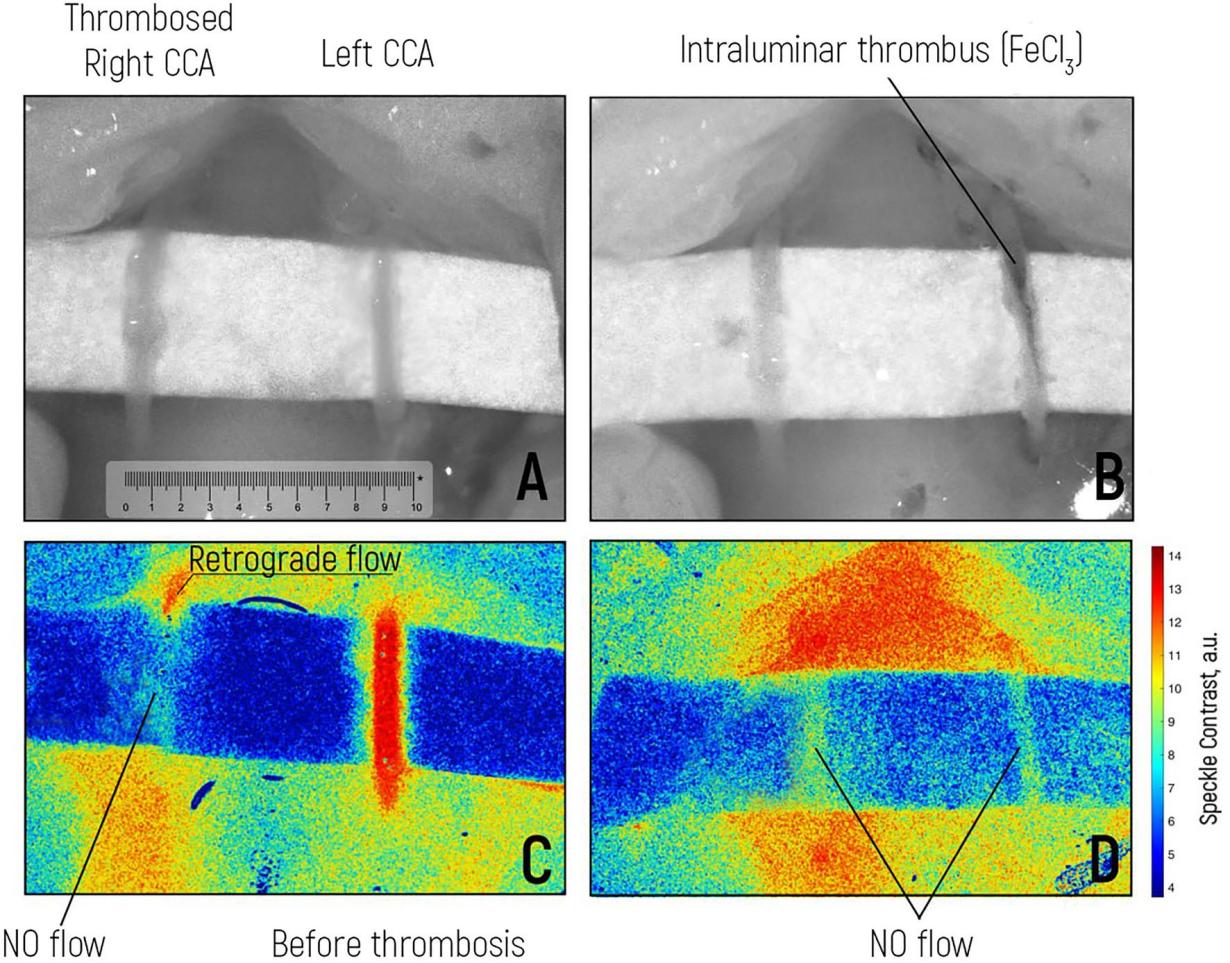
Experiment with the formation of arterial thrombosis using iron (III) chloride (FeCl3). (**A**) Intraoperative view of the right and left common carotid arteries. The right CCA was already thrombosed (control). (**B**) Intraoperative view of induced thrombosis of the left CCA with the application of iron(III) chloride: a thrombus is detected in the lumen of the vessel. (**C**) LSCI visualization of blood flow in the left CCA before thrombosis. The right CCA was thrombosed, and there was no blood flow. (**D**) Absence of blood flow according to the LSCI in both CCAs after the application of iron(III) chloride to the left CCA. * Scale bar is 10 mm.

The creation of an intraoperative model of intravascular thrombosis using FeCl3 made it possible to assess how sensitive LCSI RT is in violation of intraluminal blood flow (Fig. 4). The right CCA was used as a control. The artery was dissected through the inversion of the adventitia into the lumen of the vessel to induce intraluminal thrombosis without artefacts from movement or the instrument. The absence of blood flow was confirmed by Doppler data, which were compared with the LSCI data (Table 3). The left CCA was initially functional (see Fig. 4C). After the application of the pad containing FeCl3 solution for 2 min, a model of intraluminal thrombosis was reproduced (see Fig. 3B and D). LSCI RT confirmed the absence of blood flow after vessel thrombosis. This result is critical in the intraoperative assessment of iatrogenic intravascular thrombosis.

**Table 3:** Values of LBFV and LSCI for predicting CCA thrombosis.

	Mean value (standard deviation)	Dynamics of the average value in percents	Statistical criterion and its value	*p* value
LBFV, left CCA, proximally, initially (cm/sec)	21.13 (± 6.40)	−100.00%	Wilcoxon Test (Z) = −5.068	< 0.001
LBFV, left CCA, proximally, after FeCl3 (cm/sec)	0 (± 0)			
LSCI left CCA proximally initially (a.u.)	13.24 (± 0.23)	−58.78%	Paired Student t Test (T) = 46.070	< 0.001
LSCI left CCA proximally after FeCl3 (a.u.)	5.46 (± 0.43)			
LBFV, left CCA, distally, initially (cm/sec)	22.803 (± 1.62)	−100.00%	Wilcoxon Test (Z) = −5.145	< 0.001
LBFV, left CCA, distally, after FeCl3 (cm/sec)	0 (± 0)			
LSCI left CCA distally initially (a.u.)	13.40 (± 0.22)	−64.45%	Paired Student t Test (T) = 68.268	< 0.001
LSCI left CCA distally after FeCl3 (a.u.)	4.76 (± 0.20)			
LBFV right CCA, proximally, initially (cm/sec)	0 (0)	0.00%	–	–
LBFV, right CCA, proximally after FeCl3 (cm/sec)	0 (0)			
LSCI right CCA proximally initially (a.u.)	11.54 (± 1.23)	−50.50%	Paired Student t Test (T) = 8.793	< 0.001
LSCI right CCA proximally after FeCl3 (a.u.)	5.71 (± 0.79)			
LBFV right CCA, distally, initially (cm/sec)	0 (0)	0.00%	–	–
LBFV right CCA, distally, after FeCl3 (cm/sec)	0 (0)			
LSCI right CCA distally initially (a.u.)	7.88 (± 1.45)	−35.85%	Paired Student t Test (T) = 5.635	< 0.001
LSCI right CCA distally after FeCl3 (a.u.)	5.05 (± 0.27)			

The mean LBFV of the left CCA proximal to the initial site was 21.13 (± 6.40) cm/sec, which decreased to 0 (± 0) cm/sec after FeCl3-induced thrombosis. This finding was undoubtedly complete intraluminal occlusion. The mean LSCI of the left CCA proximal to the initial site was 13.24 (± 0.23) a.u., which decreased to 5.46 (± 0.43) a.u. after the FeCl3 reaction. The LBFV of the right CCA was initially 0 (0) cm/sec and remained the same. The LSCI of the right CCA distally was initially 7.88 (± 1.45) a.u., which decreased by −35.8%.

## Discussion

In the framework of this work, we investigated the feasibility and effectiveness of using REAL-time blood flow control via the LSCI method for simultaneous surgical procedures in an animal model. Several options for using LSCI in real time have been proposed. Moreover, these authors proposed options for integrating the LSCI system with a surgical microscope^8,11^. To date, all of these studies have been experimental.

### Analysis of the obtained results

The results of our study allowed us to say that the LSCI system allows real-time assessment of blood flow disturbance in vessels without clinically significant delays in standard situations encountered in cerebrovascular surgery, such as temporary clipping of the artery, arterial trapping, patency of microvascular anastomoses, intraoperative arterial thrombosis, or lCCAl thrombolysis. Despite the noise from movements during surgical procedures, the reliability of the data obtained on the qualitative presence or absence of blood flow does not decrease. LSCI correlates with the decrease in the LSCI according to contact Doppler sonography, and in certain situations, LSCI is a more sensitive method. The effectiveness of the method for assessing retrograde blood flow against the background of proximal occlusion is worth noting, as this method can be of great importance in the intraoperative risk assessment of ischaemic complications. Additionally, when assessing the conditional values and their dynamics during the experiments, we noticed that the relative units fluctuate within certain limits, not decreasing to absolute 0 in the absence of blood flow. In our opinion, this is due to the choice of conditional values of the LSCI scale, the noise from movement, and the peculiarities of the method. In the experiment involving vessel thrombosis, in the case of an absolute absence of blood flow velocity according to Dopplerography, the decrease in the LSCI ranged from 22.3 to 43.4%. Additionally, in the experiment with anastomosis, with a decrease in the LBFVV according to Doppler data, we observed an increase in the LBV (Table 2), which may indicate a decrease in blood flow velocity and an increase in volumetric blood flow against the background of an increase in vessel diameter after anastomosis. Systemic autoregulation of blood flow may occur after temporary clamping of the common carotid arteries and restoration of blood flow. This observation, in our opinion, is of particular interest and requires further research.

### LSCI in modern neurosurgery and its limitations

We did not find any references in the literature concerning the use of LSCI RT for vascular anastomoses, intravascular thrombosis, or lCCAl thrombolysis. Our data suggest that this method can be used in clinical practice. In our opinion, the integration of the LSCI into an intraoperative microscope is an important step, but in recent years, there have been an increasing number of publications on the use of neurosurgical exoscopes. The variant in which our work was performed implies the use of the exoscope principle. We have not observed LSCI systems integrated into a neurosurgical exoscope in the literature^1^.

The use of the LSCI system in real time has a number of limitations. The first is the quality of the data received. During movements and surgical manipulations, ROIs can shift, which leads to so-called noise and interference. Nevertheless, it is important for a neurosurgeon to qualitatively understand whether there is blood flow in a vessel after aneurysm clipping, tumor removal, or vascular anastomosis.

A stationary mode that permits the recording of correct perfusion without motion artefacts due to surgeon manipulation is established within 1 s after movement ceases. The delay of the received data is not a significant problem, as it is leveled by the frame rate per second. We can improve this parameter with a high-end camera and laser. The second problem is the limitation of obtaining data from tissues, blood or other fluid on the surface of vessels. As shown in Fig. 1, it was possible to reliably obtain data on the presence of blood flow only within the selected part of the CCA. We could not receive a signal from the blood flow through the soft tissues. The soft tissues themselves undergo perfusion, which is visible on the LSCI color scale. This can make it difficult to reliably determine the presence of blood flow with incomplete clamping or retrograde flow. We used a sterile latex lining to facilitate manipulation of the vessels, which also allowed us to reduce the signal from the tissues surrounding the vessels. This approach made it possible to improve the visualization during the experiment. We can also reduce the tissue perfusion color threshold to visualize only tissues with the highest LSCI, which are arteries. It is possible to limit the ROI with visualization of the signal only from it and its overlay on intraoperative images in white light (see Fig. 2).

Another problem is the difficulty of visualizing vessels with low blood flow, such as veins. However, some authors were able to visualize venous blood flow well using appropriate algorithms^11^. Additionally, Duncan et al. proposed an algorithm for processing LSCI images with the ability to determine the direction of blood flow, which can be useful for determining veins and arteries^14^. However, the authors state that this method requires further development and thus far has a number of errors. In the presence of bleeding or blood clots over vessels, the quality of the data obtained from the LSCI is reduced. Moreover, the presence of glare or additional light sources can degrade the image.

### Advantages and prospects of LSCI

Despite the limitations of the method, it undoubtedly has significant advantages. The LSCI system provides the option of avoiding the use of contrast medium injection and the insertion of measuring probes into the operating field. The system quickly responds to changes in blood flow in the vessel and provides reliable information in a timely manner, which in some cases is critical. For example, when using fluorescence, the injection of a contrast agent (e.g., ICG or fluorescein) takes several minutes. Video angiography can be repeated only after 15–20 min, when the contrast from the previous study leaves the vascular bed^15^. We compared the LSCI to intraoperative contact Doppler ultrasonography. Contact Dopplerography is a noninvasive diagnostic method that uses sound waves to produce images of blood moving through arteries. It is a method of manual diagnostics characterized by a certain inaccuracy. The results depend on the angle of insonation of the vessel, the point of investigation, the thickness of the vessel and other variables, which qualifies the technique as more qualitative than quantitative.

The LSCI system is safe, as confirmed by several experimental studies, and has no risk of complications1. However, real-time LSCI has not yet been applied in neurosurgical practice in a large cohort of patients. We are confident that promising areas for further research using LSCI in neurosurgical practice include the integration of LSCI into surgical exoscopy, microscopy, endoscopy and noise correction using artificial intelligence algorithms. Additionally, objective data on blood flow characteristics, such as the direction and linear velocity of blood flow, volume flow and vessel wall thickness, were obtained using the LSCI method in real time.

## Study limitations

The main limitation of the study is that it is based on animal models, and as a result, its results cannot form the basis for direct clinical recommendations. It is also worth noting that the study was performed on a small number of animals, which may affect the statistical reliability of the information obtained. Nevertheless, we believe that the results of the experiment are an important addition to the existing knowledge about the problem and define the main vectors in which further research should be carried out.

## Conclusion

The real-time LSCI method is a promising method for use in neurosurgical practice. This approach allows timely diagnosis of intraoperative disturbance of blood flow in vessels in cases of clip occlusion or thrombosis. Additionally, LSCI allows us to reliably confirm the functioning of the anastomosis and reperfusion after removal of the clips and thrombolysis in real time. An unresolved limitation of the method is noise from movements, but this does not reduce the value of the method. Additional research is required to improve the quality of the data obtained.

## Materials and methods

### Animals

The study protocol was approved by the lCCAl ethical committee of Sechenov University (protocol no. 012/22 date 04.02.2022). Our study methodology and reporting strictly adhered to the ARRIVE guidelines as described in 'PLoS Biology 8(6), e1000412, 2010′^16^. This adherence includes comprehensive details on animal handling, experimental procedures, and welfare considerations. Furthermore, we confirm that all methods of anaesthesia and euthanasia employed in this study are consistent with the commonly accepted norms of veterinary best practice. In particular, we followed the guidelines outlined in the American Veterinary Medical Association (AVMA) Guidelines for the Euthanasia of Animals (2020). These guidelines serve as our reference for ensuring the highest standards of care and ethical treatment of animals involved in our research. We have avoided the use of substances such as chloral hydrate, ether, and chloroform, which are not in alignment with current best practices for anaesthesia or euthanasia. Male 2-month-old Wistar rats weighing 250–300 g (n = 9) were used for the experiments. Each experiment that included an assessment of blood flow in various surgical procedures (group 1—model of stenosis, proximal occlusion and CCA trapping; group 2—vascular microanastomosis; and group 3—intraoperative thrombosis) was repeated 3 times on each rat in the group (n = 3 in a group). The baseline blood flow in corresponding vessels with no surgical intervention served as the control in our experiment. The sample size was chosen as the minimal number of animals (in accordance with the 3R principle) enough for the statistical assessment of the obtained results. The animals were included and randomized by veterinarians in groups in which body mass was the leading criterion (not more than a 10% difference). After 2 weeks of quarantine, the veterinarian evaluated the health of the animals and excluded those with signs of any kind of disease in accordance with GLP principles. After the quarantine, no animals were reported to be excluded. The animals were stored in a separate room in standard cages (3 animals in a cage) under controlled environmental conditions controlled by temperature (22–24 °C), humidity (45–65%), bacterial purity and air exchange per hour (not less than 10 times/hour), with limited access to personnel to minimize potential influence. The experiment was conducted by animal surgery specialists with the veterinarian control of the animal statement, while the data were recorded and analysed by the engineers without providing information about the groups or possible results. We used combined anaesthesia comprising tiletamine/zolazepam (Zoletil 100, Virbac, France) at a dose of 20 mg/kg + xylazine (Xyla, Interchemie, Netherlands) at a dose of 5 mg/kg intraperitoneally. With a decrease in the depth of anaesthesia (in response to a painful stimulus), an additional injection of Zoletil 100 (10 mg/kg) was administered intraperitoneally. All animals were fixed on the table in the supine position. The experiments were carried out on the common carotid arteries of the rats, which approximately corresponded to the size of the cerebral arteries (1 ± 0.1 mm). There was wide access to both CCAs on the neck. Both CCAs in the rats were isolated over a wide area. A thin lining was placed under the vessels to better visualize both CCAs. For hypothesis testing of the sample size, we assessed cerebral blood flow (a.u.) as the primary outcome measure.

### Design and characteristics of the system for conducting real-time LSCI

For the experiments, the developed layout of the LSCI setup was used (see Fig. 1). This system included a CS505MU monochrome camera (Thorlabs, Inc., USA) with an MVL50M23 camera lens (Thorlabs, Inc., USA) and a laser diode (ATC; Semiconductor Devices, Russia) with a wavelength of 802 nm and an output power of 100 mW. The camera exposure time in all the experiments was 5 ms. The laser radiation was scattered using a special scatterer, ED1-C20 (Thorlabs, Inc., USA). A film polarizer was installed in front of the camera, which removed the radiation reflected from the surface of the object since it did not carry information about the movement of erythrocytes. An FB800-10 bandpass filter (Thorlabs, Inc., USA) was also installed in front of the camera to reduce the effect of background lighting.

During the studies, speckle images were recorded using standard ThorCam software (Thorlabs, Inc., USA) with a frame rate of 35 fps at full camera resolution (2448 × 2048 pixels) in postprocessing mode. In the next step, the data were processed using an algorithm developed in the MATLAB environment (MathWorks, USA). For real-time visualization, we developed a separate software package. LSCI image processing was performed using standard Fiji/ImageJ software, as detailed in Schindelin et al. (2012)^17^. This approach was adopted and verified for brain imaging by Kalchenko et al. (2015)^9^. This version provides an LSCI-map calculation with a 5 fps frame rate. In both processing modes, the speckle contrast was defined as the ratio of the standard deviation of the brightness of adjacent pixels to the average intensity in accordance with the expression:

1 $$K_{n} = \frac{{\sigma_{n} }}{{\left\langle I \right\rangle_{n} }},$$

where σ_n_ is the standard deviation of the intensity, *I* is the average intensity, n is the calculation window size, and $\left\langle {...} \right\rangle$ is the averaging operator. To reduce noise, LSCI perfusion maps (Fig. 1A–F) were averaged over 20 consecutive frames for better visualization during the presentation of the results. In this paper, the speckle contrast is calculated in a moving window of 7 × 7 pixels. Then, based on the *K* value, the relative blood flow velocity (RBF) was calculated as *1/K* measured in arbitrary units (a.u.).

Relative blood flow (RBF) score based on the LSCI. The threshold scale on the presented speckle contrast images was selected for optimal visualization of blood flow at all stages of the experiment. The same threshold was chosen for all vessel images so that they could be visualized as clearly as possible against the background of surrounding tissues. Based on the RBF values in all pixels in a given window and in accordance with the color scale, a color picture was built, demonstrating the intensity of blood flow in various parts of the arteries. Blood flow measurements were taken at points of interest (ROIs) 40 × 40 pixels, which varied depending on the nature of the experiment. For the data analysis, 3 points were identified from which information was obtained for the analysis of LSCI patterns (see Fig. 2C): 1—right CCA (control), 2—left CCA distal to occlusion, and 3—left CCA proximal to occlusion. Use of Doppler. In parallel, linear blood flow velocity (LBFV) was also measured at similar points (1, 2, and 3) using contact Doppler. In our study, blood flow was assessed using a DWL Multidop T digital transcranial Doppler ultrasonography system (Compumedics, Germany) coupled with a 16 Hz probe for cerebral blood flow velocity assessment. Standardized validation procedures were followed in line with prior studies using this system. with a 16 Hz transducer on a DWL Multidop apparatus. The data from the two methods were subsequently compared.

Assessment of blood flow in stenosis, proximal occlusion and CCA trapping. In this experiment, the effectiveness of the LSCI technique was evaluated by detecting a decrease in blood flow against the background of stenosis, proximal occlusion, or trapping of the artery. For proximal occlusion and stenosis, vascular clips were used to temporarily clamp the arteries. During stenosis, the lumen of the vessel was partially blocked in the proximal region by clips and completely blocked during occlusion (see Fig. 1) (real-time video of the experiment: link). During trapping, two vascular clips were placed on the proximal and distal parts of the left CCA. Blood flow was measured first in the normal state and then distal to the occlusion/stenosis or in the switched-off segment of the left CCA (with trapping).

Additionally, after proximal occlusion, blood flow was measured after restoration (removal of the clip) to assess reperfusion. During proximal clipping, data on blood flow distal to the cut-off by Doppler and LSCI were compared to assess the sensitivity of these methods in detecting retrograde blood flow.

Evaluation of blood flow during vascular microanastomosis. The purpose of this study was to evaluate the ability of LSCI to verify the safety of blood flow after the imposition of vascular microanastomoses (to confirm patency). A side-to-side anastomosis was used as a model. Anastomosis was performed between two CCAs after preliminary preparation according to the standard technique^13^. Blood flow was assessed first under normal conditions. Then, after the anastomosis was applied, blood flow in the left CCA distal to the anastomosis zone was assessed after preliminary clipping in the proximal region. The data before and after the imposition of the anastomosis were subsequently compared.

Evaluation of blood flow in intraoperative thrombosis patients. Intraoperative arterial thrombosis is a frequent intraoperative complication in cerebrovascular surgery. To simulate this situation, we performed an arteriotomy of the right CCA via inversion of the adventitia into the lumen of the vessel. Then, the arteriotomy was closed by two nonabsorbable 10-0 nylon sutures. Blood flow was assessed by Doppler and LSCI. Thrombosis of the right CCA was confirmed. The continuous flow parameters of the left CCA were assessed before thrombosis. The left CCA was generated by applying an iron(III) chloride-induced thrombosis model. The presence of a thrombus was verified visually, according to the microscopic picture, as was the absence of blood flow according to contact Dopplerography and LSCI.

The measurements carried out during the experiment were recorded on the map of the course of the experiment in digital medium. After the completion of the experiment, the analysis of the primary data was carried out with the calculation of the studied indicators. The numerical primary data were subsequently entered into an Excel spreadsheet for subsequent statistical processing. The statistical analysis of the experimental results was carried out using the software packages Origin, MATLAB, and Fiji.

## Statistical analyses

Statistical data processing was carried out using IBM® SPSS® Statistics Version 23.0.0.0 (SPSS, Inc., USA). The normality of the sample distribution was determined by the Kolmogorov‒Smirnov test. Significant differences were defined with the help of the chi-squared test (including Yates correction or the Mann‒Whitney U test), which depends on the characteristics of the experimental variable. A paired t test was used to compare preocclusion and postocclusion measurements within each modality for both the LSCI and LBFV. The same statistical method was also used to assess differences between distinct regions of interest (ROIs). A *p* value exceeding 0.05 was considered not statistically significant. Unless otherwise indicated, all the results are presented as the means ± standard deviations.

### Supplementary Information

Supplementary Video 1.

Supplementary Information 1.

## Data Availability

The datasets generated and/or analysed during the current study are available in the repository, https://1drv.ms/f/s!AmNlRMtkMjGrgtZv-54XcCQCcq55MQ?e=cBlHa2.
