# Supplementary material for: Real-time laser speckle contrast imaging for intraoperative neurovascular blood flow assessment: animal experimental study
Source: Sci Rep. 2024 Jan 19;14:1735. doi: 10.1038/s41598-023-51022-2 (PMC10799050; doi:10.1038/s41598-023-51022-2)
Supplement: Supplementary file 2 — Supplementary Information 1. [file 41598_2023_51022_MOESM2_ESM.docx]

*Video:*

*Sequential measurements of the parameters of the vascular blood flow of the common carotid artery using the LSCI system, in order: normal blood flow, blood flow under conditions of proximal occlusion , restoration of blood flow, blood flow under conditions of bilateral clamping of the artery by vascular clips, restoration of distal blood flow Complete recovery after vessel trapping.*
